# Programming of cardiometabolic health: the role of maternal and fetal hyperinsulinaemia

**DOI:** 10.1530/JOE-21-0332

**Published:** 2022-03-08

**Authors:** Antonia Hufnagel, Laura Dearden, Denise S Fernandez-Twinn, Susan E Ozanne

**Affiliations:** 1 University of Cambridge Metabolic Research Laboratories and MRC Metabolic Diseases Unit, Wellcome Trust-MRC Institute of Metabolic Science, Level 4, Addenbrooke’s Hospital, Cambridge, Cambridgeshire, UK

**Keywords:** developmental programming, fetal hyperinsulinaemia, maternal hyperinsulinaemia, gestational diabetes, pregnancy

## Abstract

Obesity and gestational diabetes during pregnancy have multiple short- and long-term consequences for both mother and child. One common feature of pregnancies complicated by maternal obesity and gestational diabetes is maternal hyperinsulinaemia, which has effects on the mother and her adaptation to pregnancy. Even though insulin does not cross the placenta insulin can act on the placenta as well affecting placental growth, angiogenesis and lipid metabolism. Obese and gestational diabetic pregnancies are often characterised by maternal hyperglycaemia resulting in exposure of the fetus to high levels of glucose, which freely crosses the placenta. This leads to stimulation of fetal ß-cells and insulin secretion in the fetus. Fetal hyperglycaemia/hyperinsulinaemia has been shown to cause multiple complications in fetal development, such as altered growth trajectories, impaired neuronal and cardiac development and early exhaustion of the pancreas. These changes could increase the susceptibility of the offspring to develop cardiometabolic diseases later in life. In this review, we aim to summarize and review the mechanisms by which maternal and fetal hyperinsulinaemia impact on (i) maternal health during pregnancy; (ii) placental and fetal development; (iii) offspring energy homeostasis and long-term cardiometabolic health; (iv) how interventions can alleviate these effects.

## Introduction

Around 50% of women worldwide enter pregnancy overweight or obese (Hill *et al.* 2019). Maternal obesity is the main risk factor for the development of gestational diabetes mellitus (GDM) in pregnancy, which affects approximately 15% of pregnancies (Guariguata *et al.* 2014). These pregnancies not only carry a higher risk for further complications such as macrosomic infants, preeclampsia and stillbirth but children born to such pregnancies are at increased risk of poor cardiometabolic health (Dashe *et al.* 2009, HAPO Study Cooperative Research Group 2010). The observation that an adverse *in utero* environment programmes the long-term health of the offspring, called developmental programming, is now well-evidenced with a lot of data reporting increased risk of obesity, cardiovascular diseases and type 2 diabetes (T2D) in the offspring from obese and/or GDM mothers (Godfrey *et al.* 2017). Additionally, women with GDM are more likely to develop long-term metabolic impairments such as T2D after a GDM-complicated pregnancy (Dickens & Thomas 2019).

**Figure 1:**
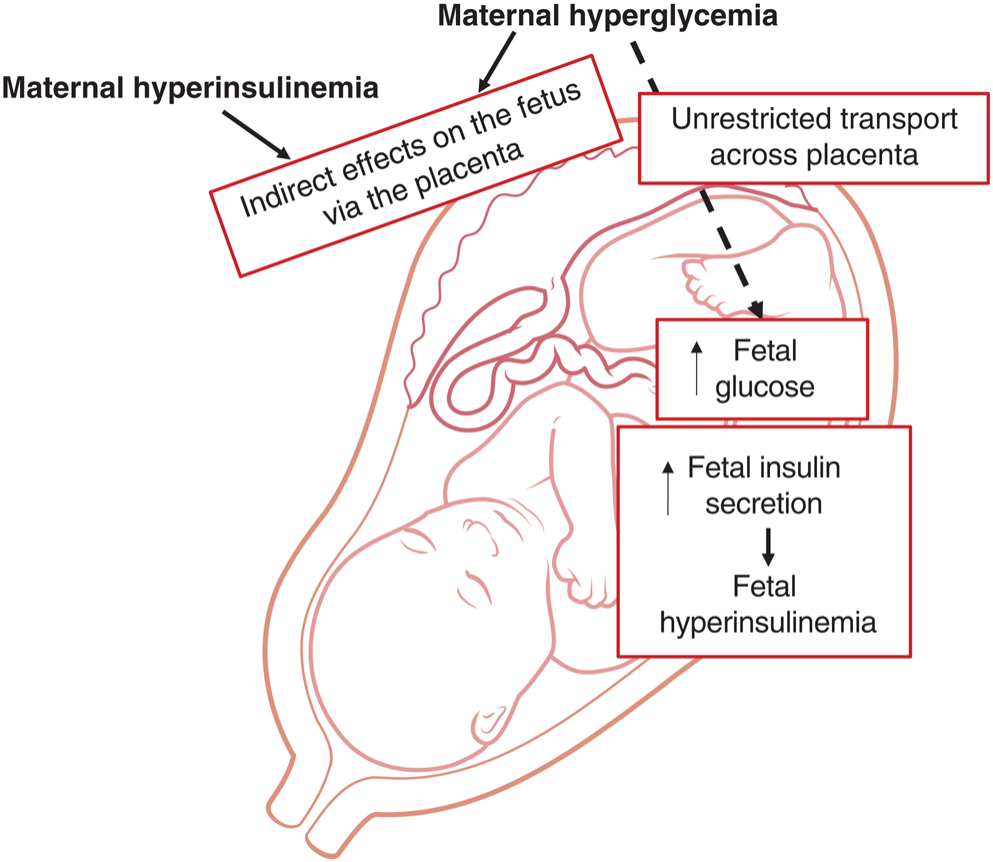
The effect of maternal hyperinsulinaemia and hyperglycaemia on the fetus. Glucose and insulin can act on the placenta and thereby indirectly influence fetal development. Insulin cannot cross the placenta; however, glucose can pass through the placenta leading to increased glucose levels in the fetus and resulting in fetal hyperinsulinaemia.

Maternal obesity and GDM are closely intertwined, with both characterised by metabolic derangements such as hyperglycaemia, inflammation, hyperinsulinaemia, hyperleptinaemia and dyslipidaemia in the mothers (Scifres *et al.* 2014, Plows *et al.* 2018). All of these factors could mediate the effects of the maternal metabolic state on offspring health. Maternal hyperinsulinaemia, a common feature in obese and GDM pregnancies, is a promising candidate that could mediate adverse effects. Although insulin does not cross the placenta, insulin receptors are expressed on the placenta and therefore insulin can mediate effects through action on the placenta (see below). Furthermore, obese and GDM pregnancies are associated with hyperglycaemia. Glucose freely crosses the placenta into the fetal circulation and stimulates the release of insulin from fetal pancreatic ß-cells, thus causing fetal hyperinsulinaemia. Both the mother and the fetus are therefore hyperinsulinaemic under such conditions as highlighted in Fig. 1.

Insulin has been shown to have effects at various stages of development. *In vitro* treatment with high insulin levels impairs early blastocyst development (Chi *et al.* 2000) and high maternal insulin levels in early pregnancy have been linked to increased placental growth (O’Tierney-Ginn *et al.* 2015). Insulin is a key fetal growth determinant (Fowden 1992) and this can explain the high risk of macrosomia in babies exposed to a diabetic environment. GDM is the most common reason for hyperinsulinaemia and hyperglycaemia during pregnancy. However, insights from other diseases characterised by maternal hyperinsulinaemia such as polycystic ovary syndrome and T2D add to our understanding of the aetiology and impact of maternal and fetal hyperinsulinaemia. In this review, we have gathered current evidence for the effects of maternal and fetal hyperinsulinaemia on developmental programming and describe the direct effects of insulin on the placenta and the maternal metabolic and cardiovascular adaptation to pregnancy. We also discuss short- and long-term effects on fetal organs, particularly the heart and brain which are strongly implicated in mediating the effects of exposure to hyperinsulinaemia on long-term cardiometabolic outcomes.

## Maternal adaptations to pregnancy and hyperinsulinaemia

### Metabolic adaptations

**Figure 2:**
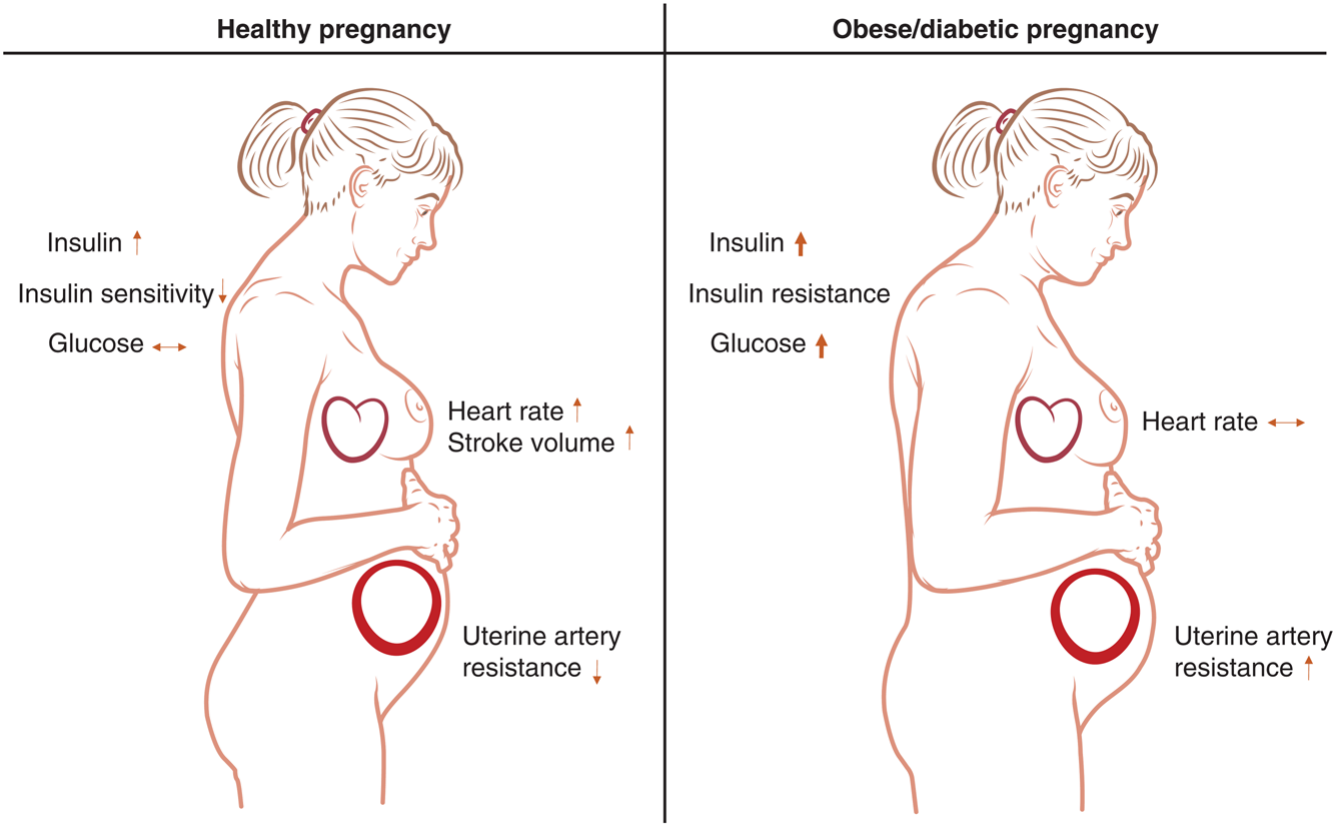
Effects of diabetes/obesity on the maternal adaptation to pregnancy. Physiological and pathological changes in pregnancy are summarised.

Drastic changes in maternal physiology are necessary during pregnancy to support fetal growth and development. In early pregnancy, metabolism adapts to increase maternal energy stores by increasing lipogenesis and glucose uptake into adipose tissue which is driven by an increase in maternal insulin sensitivity (Grimes & Wild 2018, Plows *et al.* 2018). At the end of the second trimester of pregnancy, placental hormones induce maternal insulin resistance which increases glucose production and break down of fat stores in the mother, thus releasing both glucose and fatty acids from the maternal circulation to be readily taken up by the rapidly growing fetus (Grimes & Wild 2018) (Fig. 2). It was debated up until the 1960s whether the diabetic state in the mother during pregnancy is physiological or pathological (Brown & Wyckoff 2017). It is now established that compensatory hypertrophy and hyperplasia of the pancreatic ß-cells in the mother is a normal and physiological pregnancy adaptation required to maintain glucose homeostasis which fails in pregnancies with GDM (Plows *et al.* 2018).

In obese women, impaired insulin signalling and/or pancreatic ß-cell dysfunction predispose to the development of GDM. Even before diagnosis of GDM, higher insulin levels can be detected early in pregnancy (week 16) in women who are later diagnosed with GDM (Bitó *et al.* 2005). However, comparatively little is known about insulin levels in pregnancies affected by GDM as screening focuses only on glucose levels during an oral glucose tolerance test (OGTT) at 24–28 weeks gestation. Thereby insulin levels outside the normal range are not normally picked up even though they could be beneficial for early detection of adverse outcomes or later development of GDM (North *et al.* 2021). This is supported by evidence in non-pregnant individuals suggesting that hyperinsulinaemia is a driving and initial factor in the development of metabolic diseases (Erion & Corkey 2017).

### Cardiovascular adaptations

In addition to metabolic adaptations, pregnancy also requires changes in the maternal haemodynamic system to ensure sufficient oxygen supply to the fetus (Fig. 2). By week 5 of human gestation, maternal heart rate increases (Burke 2014), then the uterine artery resistance drops at around 20 weeks gestation (James *et al.* 2017) and cardiac output increases up to 50% at week 32 of gestation (Mecacci *et al.* 2020). Impaired haemodynamic adaptations are observed in obese and diabetic pregnancies and are a major cause of complications for the mother and fetus. In 1987 Airaksinen et al demonstrated that the increase in heart rate is blunted in diabetic women (Airaksinen *et al.* 1987). A detrimental consequence of impaired vascular adaptations to pregnancy is preeclampsia. The exact pathogenesis is poorly understood but a failure of uterine spiral artery remodelling leading to increased uterine artery resistance and placental damage is thought to be important. Links can be found between insulin resistance and preeclampsia and it is thought that the signalling of insulin and vascular endothelial growth factor (VEGF), an important factor for placental vasculogenesis, converge via the phosphatidylinositol-3-kinase pathway which is impaired in insulin resistance. This pathway is involved in the activation of endothelial nitric oxide synthase (eNOS) and thereby NO production which is critical for angiogenesis and vasodilation (Scioscia *et al.* 2015). It has been hypothesised that insulin resistance and sustained hyperinsulinaemia could directly lead to endothelial dysfunction and preeclampsia (Scioscia *et al.* 2014). In the non-pregnant state, insulin resistance has been linked to endothelial dysfunction (Muniyappa & Sowers 2013) and insulin-sensitizing interventions such as exercise and metformin have been shown to improve endothelial function. Similarly, metformin treatment that leads to decreased insulin levels is thought to prevent the development of preeclampsia (Cluver *et al.* 2019). However, the observation that T1D pregnancies also have an increased risk of preeclampsia despite the lack of maternal hyperinsulinaemia highlights the complexity of the disease. The pathogenesis of preeclampsia is poorly understood and likely to be a result of multiple risk factors and placental and maternal cardiovascular impairments (Sones & Davisson 2016). Common features of GDM and T1D, that is, maternal hyperglycemic episodes and fetal hyperinsulinaemia could thereby explain the increased preeclampsia risk in both diseases.

The effects of hyperinsulinaemia on maternal haemodynamic adaptations could explain the increased risk of stillbirth in obese and diabetic pregnancies (Poston *et al.* 2016). They could also explain the cases of intrauterine growth restriction (IUGR) in these pregnancies, that are well known to be associated with a high risk for cardiometabolic diseases in the offspring (Gluckman *et al.* 2008). In addition to long-term effects on the baby, women who had preeclampsia have an increased risk of later cardiovascular diseases (Leon *et al.* 2019). On the other hand, maternal hyperinsulinaemia together with maternal hypergycaemia leads to increased glucose levels in the fetus which stimulates fetal insulin production and in turn stimulates fetal growth. This leads to macrosomia in the babies which is associated with the same long-term risks for the offspring as IUGR (Nordman *et al.* 2020).

## Placental responses to hyperinsulinaemia

### Glucose handling

**Figure 3:**
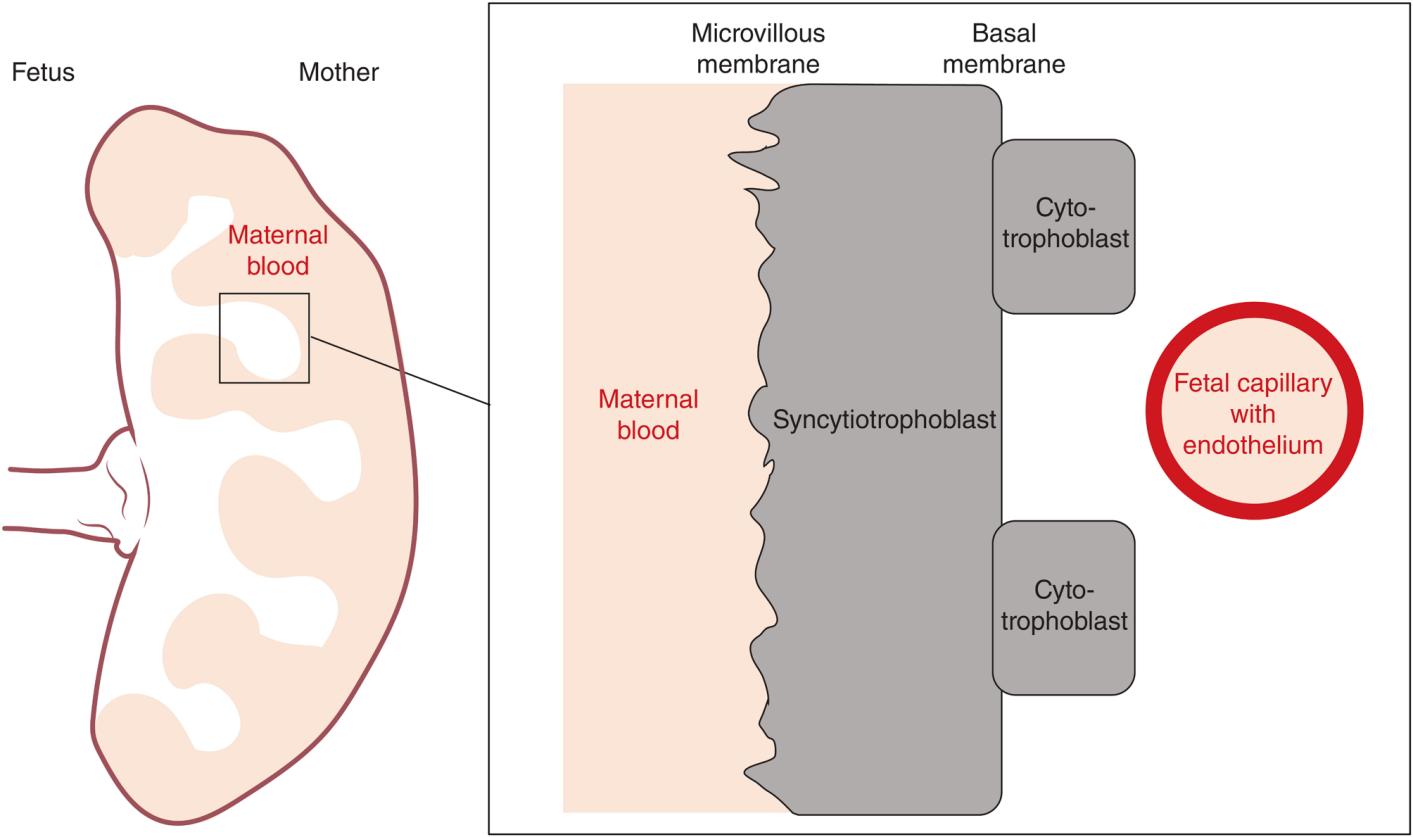
The human placenta and its barriers between maternal and fetal circulation. The villous structure of the human placenta is shown and zooming in highlights the different cellular barriers between maternal and fetal blood (adapted from Rossant & Cross 2001).

As the interface between mother and developing fetus, the placenta is key in mediating the maternal environment to immediate effects on the fetus and long-term programmed effects in the offspring after birth (Fig. 3). Despite increased overall interest and research on the effects of obesity and diabetes in pregnancy, knowledge regarding the effects of insulin on the placenta remains limited. Insulin does not cross the placenta, however, insulin receptors are abundantly expressed in the placenta, highlighting that insulin may have direct effects on it. However, the exact localisation of the receptors is debated. Mainly investigated during the 1970s, researchers showed insulin receptors associated with the glycocalyx region of the microvilli facing the maternal circulation in the human placenta at term (Nelson *et al.* 1978). The glycocalyx, a network of glycoproteins, is present on the maternal microvillous membrane and has been proposed to play a role in the regulation of transport across the syncytiotrophoblast (Fabre-Gray *et al.* 2018). In 1994 Desoye* et al*. reported that insulin receptor protein expression shifts from the maternal facing side of the placenta (the syncytiotrophoblast) to the fetal facing side of the placenta (the fetal endothelium) with advancing gestation in humans (Desoye *et al.* 1994). This led to the hypothesis that the placental response to insulin transitions with advancing gestation from insulin produced by the mother to the insulin produced by the growing fetus, and this is supported by human studies showing that insulin does not cross the placenta (Bishop *et al.* 2019). Recently, however, it was reported that insulin receptor protein expression is equally high in the maternal-facing microvillous membrane of the syncytiotrophoblast in early and late gestation compared to the fetal-facing basal membrane in the human placenta (James-Allan *et al.* 2019). Little is known about the insulin receptor localisation in placentas from non-human species. Forty to 50 years ago researchers showed that the rat placenta has significantly less insulin receptors compared to the human placenta (Testar *et al.* 1985) and that binding of insulin to the placenta is lower for rabbit, rat and mouse placentas compared to guinea pig, monkey and human placentas (Posner 1974).

Another important question relates to the functional consequences of insulin action on the placenta. In 1953, incubation of human placental slices in an insulin-enriched medium resulted in an accelerated glucose utilisation (Villee 1953). However since then conflicting results on the effect of insulin on glucose uptake in the placenta in humans and rodents have been reported (Posner 1974, Herrera *et al.* 1985, Acevedo *et al.* 2005). Glucose transport in the placenta is facilitated via carrier-mediated diffusion. In the mammalian placenta, Glucose transporter 1 (GLUT1), also known as solute carrier family 2, facilitated glucose transporter member 1 (SLC2A1) and Glucose transporter 3 (GLUT3), also known as solute carrier family 2, facilitated glucose transporter member 3 (SLC2A3) are the major transporters. High amounts of glucose transporters are expressed on the maternal-facing microvillous membrane of the syncytiotrophoblast of the human placenta, suggesting a high glucose demand for the placenta itself (Burton *et al.* 2016). In 1998 it was shown that the insulin-sensitive GLUT4 protein was detectable in both the rodent and the human placentae, but the expression levels were not changed in placental samples from diabetic women (Xing *et al.* 1998). Insulin has been shown to stimulate GLUT4 trafficking from intracellular vesicles to the syncytiotrophoblast basal membrane of the human placenta (James-Allan *et al.* 2019) but reports suggest that insulin only increased glucose uptake into the placenta in the first trimester and not at term (Ericsson *et al.* 2005). Consistent with that observation, GLUT4 expression was highest in the first trimester, whereas GLUT1 became the dominant transporter at term (Ericsson *et al.* 2005). This fits with the notion that insulin receptor localisation migrates towards the fetal side at term with maternal insulin having less of an impact in late gestation (Illsley & Baumann 2020).

### Transcriptional activation

In addition to regulating glucose transport, there is also evidence that insulin regulates transcription in first trimester trophoblasts and endothelial cells but not term trophoblast cells (Hiden *et al.* 2006). The insulin-induced transcriptomic changes did not overlap with those observed in diabetic placentas highlighting that insulin alone has different effects on diabetes, suggesting differential effects of insulin in healthy and GDM pregnancies. In a transcriptomic analysis of early trophoblasts, it was shown that cells from obese women were 30-fold less sensitive to insulin compared to cells from lean women (Lassance *et al.* 2015). This shows that the placenta can display signs of reduced insulin sensitivity when exposed to an obese environment.

### Vascular remodelling

Insulin is also thought to affect the fetoplacental vasculature with placentas from diabetic pregnancies showing hypervascularisation (Cvitic *et al.* 2014). Fetal insulin is thought to act on the fetal endothelial cells of the placenta leading to increased transport of l-arginine, a NO precursor and activation of the PI3K/Akt pathway leading to NO production and vasodilation (Pardo *et al.* 2019). Additionally, fetuses from diabetic and obese pregnancies often show hypoxia which can also stimulate placental angiogenesis and vascularisation (Cvitic *et al.* 2014). In pregnancies with pregestational maternal obesity, this vasodilatory response to insulin in the fetoplacental vasculature is impaired. A state of maternal insulin resistance might drive insulin receptor abundance on vascular endothelial cells leading to insulin activation of the MAPK pathways and secretion of endothelin leading to vasoconstriction (Ruiz-Palacios *et al.* 2017).

### Lipid handling

In addition to glucose and sufficient blood supply via the fetoplacental vasculature, the fetus relies on lipid supplies, especially in the last trimester with 90% of fetal fat deposition occurring in the last 10 weeks of human gestation (Haggarty 2002). In murine gestation, this period of fat deposition is much shorter (Machado *et al.* 2001) and murine fetuses at term only have 1–2% of fat (compared to human babies having 16% of fat) (Widdowson 1950). In both species *de novo* synthesis of lipids in the placenta is not sufficient and transport across the placenta of maternal lipids is needed. Incubation of human trophoblasts with insulin and fatty acids showed an increase of placental lipid droplets (Elchalal *et al.* 2005), consistent with increased lipid content in placentas of obese women (Calabuig-Navarro *et al.* 2017). This can lead to lipotoxicity and impair placental function due to increased oxidative stress, reduced angiogenesis and inflammation (Saben *et al.* 2014). The transport of lipids across the placenta requires hydrolysis of maternal lipoproteins into fatty acids via the placental and the endothelial (EL) lipases. The presence of GDM and obesity has been shown to increase the expression of EL (Desoye & Wells 2021). After hydrolysis, the fatty acids are taken up by the placenta via fatty acid transport proteins 1 and 4, plasma membrane fatty acid-binding protein and the fatty acid translocase CD36 (Herrera & Desoye 2016). In the placenta, the fatty acids are either used for energy metabolism via β-oxidation, stored as lipid droplets (following esterification into triglycerides) or transferred across the basal membrane of the placenta into the fetal circulation. Recent *ex vivo* perfusion experiments in human placentas have shown that only a small subset of fatty acids are directly transferred across the placenta and that the majority of fatty acids transferred to the fetus originate from the mobilisation of stored fatty acids in the placenta (Hirschmugl *et al.* 2021). Analysis of lipid metabolism in placentas from obese and lean women showed a positive correlation between fatty acid transport proteins (PLIN2 and ATGL) and maternal BMI. PLIN2 and ATGL are associated with lipid droplets and ATGL is involved in their hydrolysis (Hirschmugl *et al.* 2017). Another protein involved in triglyceride hydrolysis, CGI-58, was shown to correlate with maternal insulin levels. Therefore, maternal insulin can influence lipid levels in the placenta and their mobilisation to influence placental and fetal metabolism. Increased transfer of saturated and unsaturated free fatty acids in perfusion experiments was recently observed in placentas from obese compared to lean women (Hirschmugl *et al.* 2021). Fatty acids could programme an adverse metabolic effect in the offspring (Kabaran & Besler 2015), including the long-chain polyunsaturated fatty acid docosahexaenoic acid (DHA) that is reduced in fetuses from diabetic pregnancies (Léveillé *et al.* 2018). DHA is required for fetal brain development and is thought to protect against adiposity by modulating adipocyte development (Kabaran & Besler 2015). Treating primary human trophoblasts with glucose and insulin to mimic a diabetic environment showed a reduction of SIRT-1 regulated DHA transport highlighting the potential role for maternal hyperinsulinaemia in affecting DHA transport (Mishra *et al.* 2020).

Fetal insulin itself can also act on the placenta and is thought to increase the expression of phospholipid transfer protein (PLTP) on the basal membrane of the placenta. This protein is needed for the transport of cholesterol to the fetus (Scholler *et al.* 2012). The increased expression is thought to protect the placental endothelium from excess cholesterol and together with the increased placental vascularisation mediated by fetal insulin (as described above) could reduce the risk of placental atherosclerosis development (Desoye 2018). This led to the hypothesis that fetal insulin mediates placental adaptation to an adverse *in utero* environment. This communication between fetus and placenta mediated by fetal insulin in the advanced stages of gestation is thus important as a means of prioritising the growth of the fetus especially in the face of an adverse maternal (nutritional) environment. The next section describes first the effects of early embryo development in response to maternal hyperinsulinaemia, and then the fetal response to the placental changes and direct actions of fetal insulin on key organ development.

## Effects of hyperinsulinaemia on the fetus

The effects of insulin on the fetus are broad and start in early development. It is well-described that early pregnancy loss happens in 30–50% of women with polycystic ovary syndrome (PCOS), a condition associated with hyperinsulinaemia (Jakubowicz *et al.* 2002). Insulin and IGF1 stimulate glucose uptake into the preimplantation blastocyst via IGF1R. The switch from oxidative phosphorylation to glycolysis is important for the implantation of the blastocyst. Treatment of preimplantation blastocysts with insulin and IGF1 led to downregulation of IGF1R and thereby reduced glucose uptake, which in turn triggered apoptosis (Chi *et al.* 2000). Apoptosis was seen in the inner cell mass, which is later destined to develop into the embryo proper itself. Hyperinsulinaemia can also affect the endometrial receptivity for implantation explaining the reduced fertility in obese women and women with PCOS (Schulte *et al.* 2015).

### Fetal pancreas and insulin production

In this review so far, effects have focused mainly on maternal hyperinsulinaemia. However diabetic and obese pregnancies are also associated with fetal hyperinsulinaemia (see above). In 1920 Jorgen Petersen formulated the hypothesis that maternal hyperglycaemia leads to increased glucose levels in the fetus which in turn stimulates fetal pancreatic insulin secretion leading to fetal hyperinsulinaemia (Catalano & Hauguel-De Mouzon 2011). The human pancreas develops 4 weeks after conception and the first insulin secretion can be observed between week 7 and 8 of embryonic life. From 12 weeks onwards insulin can be detected in amniotic fluid, which is a good surrogate for fetal serum insulin as shown by a strong correlation between amniotic fluid insulin at 31 weeks and cord blood insulin levels at birth (Desoye & Nolan 2016). The fetal pancreas usually does not respond to glucose in early pregnancy; however, in diabetic women, high glucose stimulates insulin secretion as early as 12 weeks of gestation (Desoye & Nolan 2016). Therefore, fetal hyperinsulinaemia is likely present before GDM diagnosis at 24–28 weeks gestation. Desoye and Nolan proposed the ‘fetal glucose steal phenomenon’ that could mask maternal hyperglycaemia (Desoye & Nolan 2016). According to this phenomenon, fetal hyperinsulinaemia drives rapid uptake of glucose into fetal tissue creating a higher glucose gradient across the placenta between mother and fetus, thereby ‘stealing’ glucose from the mother. This problem is exaggerated in mothers with hyperglycaemia and the authors therefore propose that this phenomenon may increase the risk of false-negative GDM screening results.

**Figure 4:**
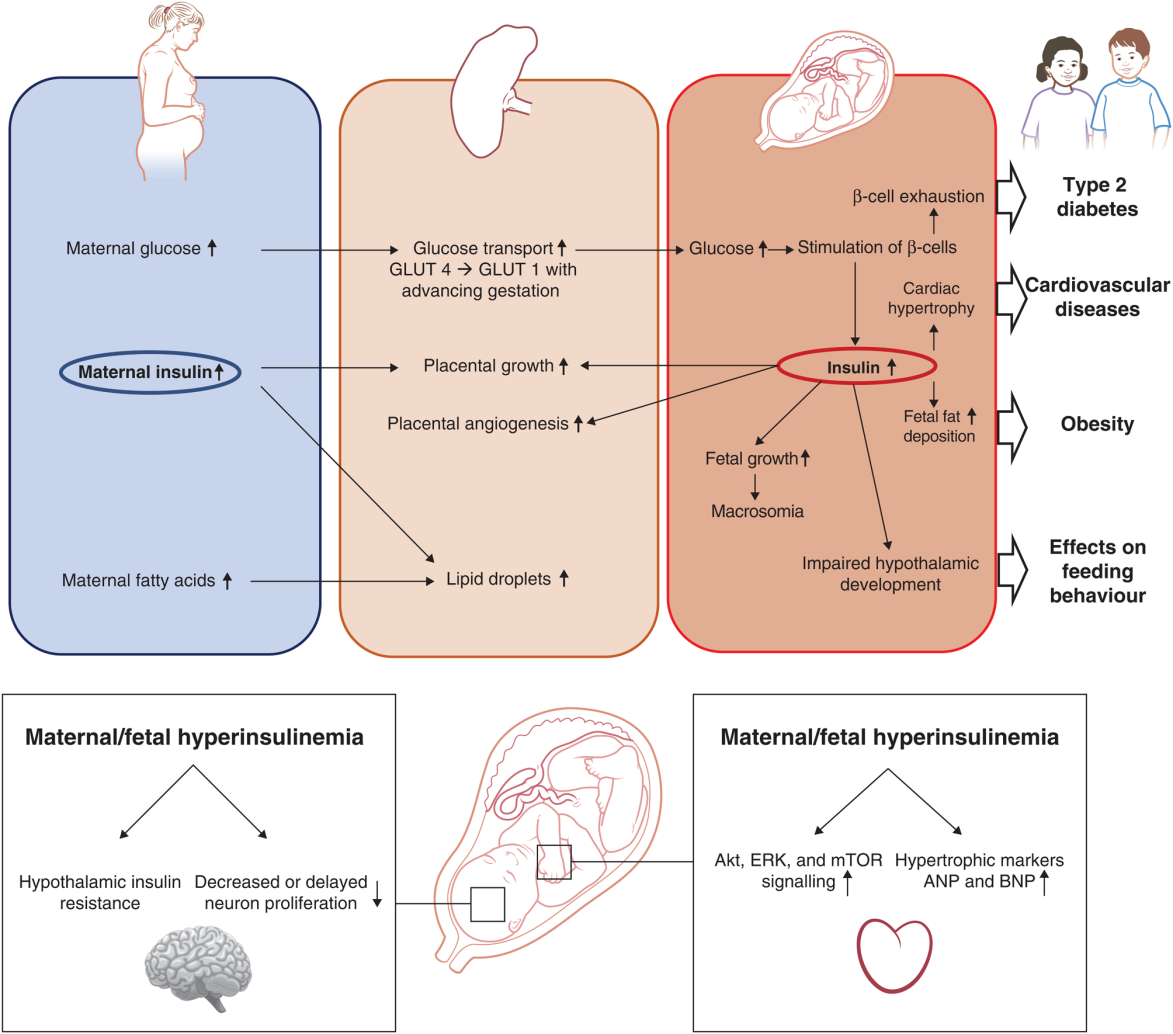
Effects of maternal and fetal hyperinsulinaemia in an obese/diabetic pregnancy.

In the mouse, the pancreas starts rapid expansion at gestational day E10.5 with insulin detectable in endocrine cells from E9.5 onwards (Jørgensen *et al.* 2007). Insulin secretion from the murine fetal pancreas can be observed from fetal pancreatic ß-cells by E15.5 (Qiao *et al.* 2019). Maternal high-fat diet feeding has been shown to lead to increased fetal pancreatic ß-cell mass as a consequence of increased placental fatty acid supply (Qiao *et al.* 2019). Similarly overfeeding in ewes caused overgrowth of pancreatic islets and ß-cell hyperplasia. This observation was made in mid-gestation around the time the progenitor cell pool for the pancreas is set (Ford *et al.* 2009). However, analysis of pancreatic tissue and pancreatic ß-cells in particular in ewes at term showed increased ß-cell apoptosis and reduced cell numbers (Zhang *et al.* 2011). It is likely that increased pancreatic ß-cell proliferation and hyperplasia occur as an initial response to compensate for hyperglycaemia; however, this leads to pancreatic ß-cell exhaustion with hypoplasia and cell death when hyperglycaemia is sustained. Evidence for this comes from experiments in sheep where pulsatile hyperglycaemia induction three times a day for one hour led to increased ß-cell numbers whereas constant hyperglycaemia for one week induced apoptosis (Frost *et al.* 2012). Thereby pancreatic development can be severely impaired in fetuses exposed to an obese and/or diabetic *in utero* environment. This early life pancreatic ß-cell deficit predisposes the offspring to the development of T2D explaining the strong association of increased risk for metabolic diseases later in life (Fig. 4).

### Insulin action on fetal growth and adiposity

As far back as 40 years ago, animal models have shown the importance of fetal insulin for fetal growth which is also highlighted in human data. Studies in twins with one twin born appropriate for gestational age and the other twin showing IUGR showed that the IUGR twins had significantly lower levels of IGF1 and insulin (Bajoria *et al.* 2002). This clearly highlights the role of fetal insulin/IGF1 for fetal growth and development in the same maternal environment. Other evidence can be found from a human study with a mutation in the glucokinase gene. This results in impaired glucose sensing by fetal pancreatic ß-cells and therefore reduced insulin secretion. Comparison of siblings with and without the mutation showed significantly reduced placental and birth weight in the sibling with the mutation (Shields *et al.* 2008). These findings are strengthened by a strong correlation between cord blood insulin levels and placental and birth weight (Shields *et al.* 2008). Notably, when the glucokinase mutation is present in the mother but not in the fetus, this leads to maternal (and consequently fetal) hyperglycaemia, fetal hyperinsulinaemia and increased fetal growth. Early studies showed that rhesus monkey fetuses infused with insulin via implanted minipumps in the last third of gestation became macrosomic (Susa *et al.* 1984). Similar results were obtained by injection of long-acting insulin into the hind leg of rat fetuses at day 21 of gestation. In addition to macrosomia, increased fat content in the insulin-treated fetuses was also observed (Angervall *et al.* 1981).

The association between fetal insulin and fetal adiposity has now been proven in human and animal studies. The Hyperglycaemia and Adverse Pregnancy Outcomes study was the first analysis that systematically assessed the relationship between maternal hyperglycaemia and adverse pregnancy outcomes to define the threshold for GDM diagnosis. A recent analysis of this data set highlighted strong associations of cord blood C-peptide levels and both neonatal and offspring adiposity (Josefson *et al.* 2021). Interestingly another study showed that the association between maternal insulin and neonatal adiposity is weaker in female neonates (Desoye & Van Poppel 2015). In cord blood, C-peptide rather than insulin is often measured due to the high levels of haemolysis in cord blood that affect insulin measurement (as it is associated with insulin degradation) but not C-peptide (Lowe *et al.* 2008). The promotion of fetal adipose tissue growth by insulin could explain the positive correlations observed between fetal insulin and fetal leptin levels in babies born large for gestational age (Wolf *et al.* 2000). This highlights the effect of sustained hyperinsulinaemia in the fetus on white adipose tissue expansion and leptin synthesis in adipose tissue. Even without macrosomia neonatal adiposity was shown to be increased in babies from mothers with GDM (Catalano *et al.* 2003). This is significant as it is well established that increased neonatal adiposity is associated with increased obesity later in life (Fig. 4).

### Insulin action on the fetal cardiovascular system

A commonly reported complication of diabetic pregnancies is stillbirth with a 14-fold higher risk compared to non-diabetic pregnancies (Lynch *et al.* 2020). It is hypothesised that stillbirth occurs due to fetal hyperinsulinaemia-driven overgrowth, excess fat deposition and increased metabolism leading to fetal hypoxia (Mathiesen *et al.* 2011). Another cause for stillbirth could be cardiac dysfunction, 40% of newborn infants from T1D women show cardiac enlargement and asymmetric hypertrophies. Insulin signalling contributes to both embryonic and postnatal cardiac growth (Bertrand *et al.* 2008). This responsiveness to insulin combined with a fetal overexposure induced by maternal obesity or diabetes may result in fetal cardiac cell overgrowth. Indeed, this relationship has been revealed in newborn infants with congenital hyperinsulinism exhibiting hypertrophic cardiomyopathy (HCM) (Huang *et al.* 2013). The role of insulin in programming a cardiac phenotype as a primary outcome is supported in mouse studies by links between maternal hyperinsulinaemia resulting from maternal obesity, and offspring cardiac hypertrophy in the young adult. The cardiac tissue from offspring of obese mouse dams was found to have increased insulin responsiveness through activation of AKT, ERK and mTOR growth signalling pathways (Fernandez-Twinn *et al.* 2012), which may also involve an activation of insulin-IGF1 hybrid receptors (Maillet *et al.* 2013) (Fig. 4).

This cardiac hypertrophy was present from as early as weaning (Blackmore *et al.* 2014) and was determined to be pathological and related to an activation of the fetal gene programme and associated with cardiac dysfunction (Fernandez-Twinn *et al.* 2012, Blackmore *et al.* 2014, Loche *et al.* 2018, van der Pol *et al.* 2020). Supporting studies in other animals have shown, for example, in rats born to mothers fed a high-fat diet, that MEF2-regulated hypertrophic markers ANP and BNP were already increased in early postnatal life (De Jong *et al.* 2018) while in sheep, cardiac hypertrophy and contractile function has been shown to be adversely affected from as early as fetal life as a consequence of maternal obesity (Wang *et al.* 2019). Cardiac hypertrophy has also been observed as early as embryonic day 17.5 in a model of maternal obesity with T2D (Lin *et al.* 2017) similar to ours (Fernandez-Twinn *et al.* 2012).

In our lab, we explored further the link between maternal hyperinsulinaemia and fetal cardiac hypertrophy using our mouse model of maternal diet-induced obesity. We showed that daily treadmill exercise was not only able to normalise pregnancy hyperinsulinaemia in the obese mother, but it also prevented offspring cardiac hypertrophy (Beeson *et al.* 2018) and suppressed several markers of the cardiac fetal gene programme and prevented offspring hyperinsulinaemia (Fernandez-Twinn *et al.* 2017).

One proposed mechanism for the link between maternal obesity-hyperinsulinaemia and offspring left ventricular remodelling and hypertrophy is maternal oxidative stress. To test this, Zhang *et al.* (2021) treated obese mice mothers with a US Food and Drug Administration-approved antioxidant N-acetylcysteine during pregnancy and found that it prevented offspring cardiac remodelling in a sex-dependent manner, and was associated with decreased activation of cardiac proliferation-related signalling molecules AKT and S6 kinase. In rats, oxidative stress generated by gestational hypoxia has also been shown to cause enhanced myocardial contractility, impaired ventricular relaxation and cardiac sympathetic dominance, which can be effectively targeted and rescued by treatment with the mitochondria-targeted antioxidant MitoQ, a drug that mimics the effects of the endogenous mitochondrial antioxidant coenzyme Q10 (CoQ10) (Spiroski *et al.* 2021).

The numerous associations between maternal hyperinsulinaemia, offspring hyperinsulinaemia and cardiac hypertrophy involving activation of insulin responsive pathways (Fernandez-Twinn *et al.* 2012), highlight the need for further study into the proposed interactions and mechanisms between maternal insulin, maternal oxidative stress and fetal cardiac development.

In humans, hyperinsulinism is widely associated with cardiac hypertrophy (CH) which cannot be distinguished from HCM on echocardiographic examination (Paauw *et al.* 2020). CH has been reported in up to 44% of infants born to diabetic mothers and in 40% of infants with congenital hyperinsulinism. Various maternal lifestyle interventions with or without dietary interventions trials in various cohorts of women at risk of GDM have now begun to explore the offspring effects on childhood adiposity, however, there is scarce information on offspring cardiovascular effects. Encouragingly, one such report has emerged in the UK Pregnancies Better Eating and Activity Trial, a randomised controlled trial of an antenatal diet and physical activity intervention in 1555 women with obesity. The study showed a positive association of the complex intervention on resting pulse rate in the follow-up of 3-year-old children (Dalrymple *et al.* 2020).

Coincident with the CH, our studies in mice have also provided evidence for systolic and diastolic dysfunction and cardiac sympathetic dominance in adult offspring of obese mothers (Blackmore *et al.* 2014, Loche *et al.* 2018). Others (Samuelsson 2014) have shown a hypertensive phenotype is developmentally programmed through exaggerated postnatal leptin. Our work using the model of maternal obesity intervened by exercise supported a divergence in the programming pathways leading to cardiac and hypertension phenotypes as hypertension is not corrected by normalising maternal and offspring hyperinsulinaemia (Beeson *et al.* 2018). These studies underline the complexity of maternal obesity in which many changes to maternal metabolism are manifest, including hyperinsulinaemia, hyperleptinaemia and hyperlipidemia. How these changes interact with the developing zygote, embryo and fetus, the mediation of the placenta and the fetus’ own response present particular challenges for future study.

### Insulin action on the fetal/neonatal brain

Offspring weight gain following exposure to hyperinsulinaemia, as in the context of an obese and/or diabetic pregnancy, is often preceded by increased food intake, implicating the brain as a major route of programme ming long-term changes in energy homeostasis (Dearden & Ozanne 2015). Gestational diabetes is associated with a wide spectrum of changes in CNS development and long-term function in exposed offspring, including neurocognitive and neurodevelopmental defects (Ornoy 2005, Moheet *et al.* 2015) and psychological disorders such as schizophrenia (Van Lieshout & Voruganti 2008). Whether these long-term effects in the offspring are caused by exposure to high levels of glucose, insulin, or both, during a diabetic pregnancy remains to be discovered in human studies. However, work in rodent models has allowed researchers to delineate some of the effects of insulin and glucose showing a pivotal role for insulin in mediating some of the deleterious effects of exposure to an obese or diabetic pregnancy on offspring brain development.

Pro-insulin mRNA is detected in fetal mouse heads at a pre-pancreatic stage (E9.5) suggesting a developmental requirement within the brain (Deltour *et al.* 1993). Brain insulin receptor expression is consistently high during neonatal life but declines during adulthood, further supporting a role for insulin in brain development (Potau *et al.* 1991, Schulingkamp *et al.* 2000). Interestingly, mice with a genetic deletion of the insulin receptor in the brain do not display any gross differences in brain development or morphology (Brüning *et al.* 2000). This is likely explained by the strong synergy between insulin and IGF signalling in the brain; as in peripheral tissues, Insulin receptors and IGF1 receptors can heterodimerize, and in the rabbit brain the majority of insulin receptors appear to exist as heterodimers (Bailyes *et al.* 1997). Thus, it is likely that in the brain, insulin and the IGFs exhibit considerable cross-talk in signalling and may compensate developmentally. Knockout of the IGF1 receptor in the brain results in reduced brain size, generalised growth retardation, and homozygous knockout is lethal at birth (D’Ercole *et al.* 2008). Similarly, knockout of IRS2, a critical downstream mediator of insulin signalling in the brain, results in a huge reduction in brain size caused by a 50% reduction in neuronal proliferation (Schubert *et al.* 2003). These mice survive to adulthood but are overweight, hyperinsulinaemic and glucose intolerant (Taguchi *et al.* 2007), demonstrating the importance of insulin signalling *in utero*for both brain development and the long-term consequences for the regulation of energy homeostasis.

Experiments in primary neuronal cultures have established a neurotrophic, neuromodulatory and neuroprotective role for insulin in neuronal cells including neurons, glia and oligodendrocytes. Insulin has been shown to stimulate axon outgrowth from neurons in culture from numerous brain regions and across a range of species (Recio-Pinto & Ishii 1984, Toran-Allerand *et al.* 1988, Fex Svenningsen & Kanje 1996, Song *et al.* 2003, Lázár *et al.* 2018). Accumulating data support the idea that insulin signalling plays a prominent role in both structural and functional aspects of synapses in the adult brain, although the detailed molecular mechanisms by which insulin controls synaptic function and dendritic structure are not known (Chiu & Cline 2010). Insulin has an important function in both embryonic and adult stem cell homeostasis across species via its role in maintaining neural stem cell self-renewal, neurogenesis, and in some instances, promoting differentiation (Vento *et al.* 1997, Ziegler *et al.* 2015). Insulin also acts to inhibit neuronal apoptosis via activation of protein kinase B and protein kinase C (Dudek *et al.* 1997, Apostolatos *et al.* 2012) resulting in increased neuronal survival.

One of the brain regions that has a high density of insulin receptors and mediates many of the important physiological functions of brain insulin signalling is the hypothalamus. The hypothalamus is essential for maintaining energy homeostasis, explaining why changes in the development of this region of the brain are associated with long-term cardiometabolic outcomes. In rats, bilateral hypothalamic insulin agar implants during the early postnatal period, when the hypothalamus is still developing in rodents, result in an increase in body weight that begins around postnatal day 21 (Plagemann *et al.* 1992) demonstrating that changes to hypothalamic insulin levels during this critical developmental period have long-lasting effects on body weight. Maternal and fetal insulin signalling is important for the correct development of neurons containing feeding-related neuropeptides in the hypothalamus. Maternal hypoinsulinaemia during pregnancy induced by streptozotocin injection increases the number of neurons expressing neuropeptides involved in energy balance regulation (such as neuropeptide Y, pro-opiomelanocortin (POMC) and tyrosine hydroxylase) in the arcuate nucleus of the hypothalamus (Plagemann *et al.* 1998a, 1998b, Steculorum & Bouret 2011). The reported increase in NPY/AgRP neuronal number is rescued by the normalisation of maternal hyperglycaemia following maternal pancreatic islet transplantation, demonstrating that the effect on the offspring’s hypothalamic morphology is caused by the change in maternal insulin or glucose levels (Franke *et al.* 2005). Moreover, an increased number of POMC neurons is found in insulin receptor deficient mice, which can partially be rescued by the specific re-expression of the insulin receptors on POMC neurons, demonstrating a cell autonomous effect for insulin signalling in determining POMC cell number in the hypothalamus (Plum *et al.* 2012). Cross-fostering experiments indicate that pups born from control mothers and raised by diabetic mothers have an altered number of arcuate neurons, showing the importance of postnatal insulin levels in influencing hypothalamic cell number (Fahrenkrog *et al.* 2004). Consistent with this idea, in rats both subcutaneous injection of insulin and hypothalamic insulin implants during the postnatal period are sufficient to induce lifelong morphological remodelling of hypothalamic nuclei, including a reduction of neuronal density in the ventromedial hypothalamus, that is associated with later obesity (Harder *et al.* 1998, Plagemann *et al.* 1999).

There is also evidence from rodent studies that insulin signalling is required both in the pre- and post-natal periods for correct development of the complex network of hypothalamic projections that control energy homeostasis. In mice, maternal insulin injections in late pregnancy result in obesity in male but not female offspring (Jones *et al.* 1995). This experimental manipulation also results in an increased innervation of the paraventricular nucleus of the hypothalamus by norepinephrine-containing fibers (Jones *et al.* 1996), suggesting that the impact of maternal insulin injections on offspring obesity may be mediated through its organizing action on feeding-related fibers in the paraventricular nucleus. In contrast, a reduction of maternal insulin levels achieved by maternal streptozotocin injection disrupts the development of key hypothalamic melanocortin circuits involved in feeding regulations (Steculorum & Bouret 2011). In addition, although the genetic deletion of insulin receptors from POMC neurons does alter their development under normal conditions, it prevents the disruption of arcuate POMC projections to the pre-autonomic compartment of the paraventricular nucleus that occurs in offspring exposed to an obese and hyperinsulinaemic pregnancy, suggesting this disrupted circuit development in response to maternal nutrition is mediated largely through insulin signalling (Vogt *et al.* 2014). It has recently been shown that the impact of insulin on the growth of primary neuronal cultures originating from the arcuate nucleus of the hypothalamus is dependent on prior postnatal nutritional status, further demonstrating an important interaction between insulin signalling and nutritional state in determining neuronal growth and circuit formation (Decourtye *et al.* 2018). Placental insulin signalling has additionally been shown to be involved in neurodevelopment as placental insulin receptor knock out in mice led to impaired serotonin signalling and synthesis in the male but not female placenta manifesting in neurodevelopmental disorders in the male offspring at eight weeks of age (Bronson *et al.* 2016).

During pregnancies complicated by maternal obesity or GDM, both maternal and fetal insulin levels are high in response to maternal hyperglycaemia. The effects of persistent hyperinsulinaemia on brain development are not well characterised; however, a study in humans has shown that fetal brain activity is altered in response to a maternal oral glucose challenge and that the level of fetal brain response is correlated with maternal insulin sensitivity (Linder *et al.* 2014). Given the neurotrophic role reported for insulin during development, it is likely that fetal brain insulin signalling is a prerequisite for appropriate brain maturation. However, chronic hyperinsulinaemia, which is present in insulin resistant mothers and corresponds to high insulin levels in the fetus, might induce insulin resistance in the fetal brain. A recent study in mice has reported that fetuses of obese, hyperinsulinaemic dams display reduced expression of proliferative genes in the hypothalamus and disrupted neural stem cell growth in primary culture and that these two markers of neuronal proliferation were correlated with maternal insulin levels (Dearden *et al.* 2020). Due to the essential role for insulin signalling in neuronal stem cell self-renewal and neurogenesis, insulin resistance in the developing hypothalamus could explain the reduced proliferative response of hypothalamic neurons in offspring exposed to an environment of energy excess (Davidowa & Plagemann 2007, Dearden *et al.* 2020). This could then result in long-term morphological changes in the hypothalamus and ultimately a lack of energy balance regulation that results in the development of cardiometabolic disease (Fig. 4).

All these short- and long-term effects of maternal and fetal hyperinsulinaemia on the offspring’s growth and cardiometabolic health, make interventions in obese and GDM pregnancies highly valuable, which will be discussed in the last paragraph of this review.

## Interventions

In recent years, screening for and treatment of GDM has resulted in significant improvements in pregnancy outcomes and infant health, reducing macrosomia, perinatal complications and stillbirth. This has been shown in a randomised clinical trial comparing lifestyle and/or insulin intervention treatment with routine clinical care in GDM pregnancies (Crowther *et al.* 2005). However, even with the achievement of optimal periconceptual HbA1c levels as a marker for maternal glycaemia in women with pregestational T1D or T2D, the risk of fetal death was still two times higher than in women without diabetes (Tennant *et al.* 2014). This highlights the limitations of current screening methods and that not only hyperglycaemia is a risk factor for adverse outcomes. In GDM pregnancies it has been shown that even with the management of GDM no normalisation of fetal adiposity can be achieved (Desoye & Nolan 2016). This may be a consequence of diagnosis generally occurring at 24–28 weeks, that could have been preceded by many months of sub-clinical differences in glucose metabolism impacting on the developing fetus. Furthermore, the focus on and ease of measuring maternal glucose means that no attention is directed towards maternal or fetal hyperinsulinaemia which (i) may not be normalised via management of maternal hyperglycaemia and (ii) does not consider that sub-GDM threshold hyperglycaemia or short maternal hyperglycemic episodes could drive fetal hyperinsulinaemia and thereby poor outcomes (Damm *et al.* 2014). A study in rats demonstrated that increased fetal weight occurred when hyperglycemic episodes were induced via glucose injection in early pregnancy, however in later pregnancy this effect could not be observed (Ericsson *et al.* 2007). This highlights the importance of the timing of the fetal hyperinsulinaemia induced by maternal hyperglycaemia on adverse outcomes, especially given that early pregnancy is a critical time window for many organ systems such as the heart and the brain. This is reflected in recent treatment advancements showing that continuous glucose monitoring reduced the incidence of LGA and neonatal hypoglycaemia in offspring from T1D women compared to those self-monitoring blood glucose (Scott *et al.* 2020). Insulin treatment has been used for almost four decades in GDM pregnancies in which lifestyle and diet interventions fail and has shown improvement regarding macrosomia rates and adverse pregnancy outcomes by increasing maternal insulin and decreasing maternal hyperglycaemia (ACOG 2019). There are also a number of oral anti-diabetic agents in use for the treatment of GDM. Glyburide, an insulin secretagogue leads to increased maternal insulin levels and thereby reduces maternal hyperglycaemia. Comparison of glyburide and insulin treatment of GDM showed increased rates of macrosomia and neonatal hypoglycaemia in glyburide-exposed fetuses (Bertini *et al.* 2005). As glyburide can cross the placenta it is thought to stimulate fetal insulin secretion which as highlighted in this review appears to be a major driver of adverse outcomes in diabetic pregnancies. Another oral agent that has seen a steep increase in use in the past years, and is nowadays the most-prescribed glucose-lowering agent, is metformin (Nathan *et al.* 2009). Metformin is currently the first-line treatment for GDM in an increasing number of countries worldwide including the UK (NICE 2015). In contrast to glyburide and insulin, metformin decreases maternal insulin levels, which could be the reason for the observed improvements regarding maternal adaptation to pregnancy such as reduction of preeclampsia risk (Cluver *et al.* 2019) also highlighted in metformin intervention studies in obese mice in our lab (Hufnagel *et al.* 2021). Metformin improved maternal metabolic health but not the obesity-induced changes in the fetus and the placenta. As metformin crosses the placenta further research is needed to address the possible direct effects of metformin on the placenta and the fetus. This is especially important in light of recent human data highlighting reduced birth weight and increased adiposity later in life in children born to metformin-treated GDM mothers compared to those treated with insulin (Tarry-Adkins *et al.* 2019), which has also been shown in follow-up studies of the offspring exposed to metformin *in utero* in our animal studies (Schoonejans *et al.* 2021).

## Conclusion and outlook

This review highlights that maternal and fetal hyperinsulinaemia play an important role in mediating pregnancy complications and programme ming cardiometabolic diseases in the offspring. Maternal hyperinsulinaemia affects maternal adaptation to pregnancy and placental metabolism. Fetal hyperinsulinaemia alters fetal growth and development of important tissues such as the heart and the brain that predispose the offspring to develop cardiac dysfunction, altered energy homeostasis, T2D and obesity later in life. The high prevalence of T2D, maternal obesity and thereby GDM in pregnancy have led to an increase in research regarding the short- and long-term effects on mother and child in these complicated pregnancies. However, the exact mechanisms underlying these effects are only slowly becoming unravelled. As shown by the controversies regarding the insulin receptor expression in the placenta that has been discussed since the 1970s it is important to understand the basics of altered factors in obese and diabetic pregnancies even better to further improve treatment and prevention of these pregnancies to improve maternal and offspring health.

## Declaration of interest

The authors declare that there is no conflict of interest that could be perceived as prejudicing the impartiality of this review.

## Funding

The work was funded by the British Heart Foundationhttp://dx.doi.org/10.13039/501100000274 (RG/17/12/33167 and PG/20/11/34957), a Wellcome Trust http://dx.doi.org/10.13039/100010269 PhD studentship (108926/B/15/Z), the Medical Research Council http://dx.doi.org/10.13039/501100000265 (MC_UU_00014/5) and a Sir Henry Wellcome Fellowship (106026/Z/14/Z).
